# Convolutional Neural Network for Multi-Category Rapid Serial Visual Presentation BCI

**DOI:** 10.3389/fncom.2015.00146

**Published:** 2015-12-02

**Authors:** Ran Manor, Amir B. Geva

**Affiliations:** Department of Electrical and Computer Engineering, Ben-Gurion University of the NegevBeer-Sheva, Israel

**Keywords:** Brain computer interface (BCI), convolutional neural networks, deep learning, Electroencephalography (EEG), P300, rapid serial visual presentation (RSVP)

## Abstract

Brain computer interfaces rely on machine learning (ML) algorithms to decode the brain's electrical activity into decisions. For example, in rapid serial visual presentation (RSVP) tasks, the subject is presented with a continuous stream of images containing rare target images among standard images, while the algorithm has to detect brain activity associated with target images. Here, we continue our previous work, presenting a deep neural network model for the use of single trial EEG classification in RSVP tasks. Deep neural networks have shown state of the art performance in computer vision and speech recognition and thus have great promise for other learning tasks, like classification of EEG samples. In our model, we introduce a novel spatio-temporal regularization for EEG data to reduce overfitting. We show improved classification performance compared to our earlier work on a five categories RSVP experiment. In addition, we compare performance on data from different sessions and validate the model on a public benchmark data set of a P300 speller task. Finally, we discuss the advantages of using neural network models compared to manually designing feature extraction algorithms.

## 1. Introduction

Brain computer interface(BCI) allows direct control over a machine by using the brain's electrical activity. Traditionally, these systems were aimed at locked-in patients, but advances in computers and algorithms have enabled BCI applications for healthy users as well. In these cases, electroencephalography (EEG), a noninvasive recording technique, is commonly used for monitoring brain activity. EEG data is simultaneously collected from a multitude of electrodes at a high temporal resolution, yielding data matrices for the representation of brain activity. In addition to its unsurpassed temporal resolution, EEG is wearable and more affordable than other neuroimaging techniques, and is thus a prime choice for practical BCI applications.

Applications for healthy subjects critically depend on the ability to decode the brain activity in response to a single recording of EEG. This is in contrary to the popular practice of averaging multiple responses to the same stimuli to increase the signal to noise ratio. Single-trial EEG data contains not only measurement noise, but also interference from on-going, task independent, brain activity which often masks the target signal. Thus, most classification methods use machine learning (ML) algorithms, to classify single-trial activity matrices based on statistical properties (Pfurtscheller et al., 2002; Müller et al., 2003; Felzer and Freisieben, 2004; Lotte et al., 2007; Blankertz et al., 2007b).

Some learning algorithms use prior knowledge, such as specific frequency-bands relevant to the experiment (Pfurtscheller et al., 2002), or brain locations most likely to be involved in the specific task (Felzer and Freisieben, 2004). For instance, the literature has robustly pointed out parietal scalp regions to display high amplitude signals in target detection paradigms. This target-related response, known as the P300 component, has been repeatedly observed approximately 300–500 ms post-stimulus (Donchin et al., 1978). Prior-knowledge based algorithms, in particular P300 based systems, are commonly used for a variety of BCI applications (Donchin et al., 2002; Wolpaw et al., 2002; Wolpaw and McFarland, 2004; Sellers and Donchin, 2006). In contrast, other methods construct an automatic process to extract relevant features based on supervised or unsupervised learning from training data sets.

Approaches for automatic feature extraction include Common Spatial Patterns (CSP), auto-regressive (AR) models and Principal Component Analysis (PCA). CSP extracts spatial weights to discriminate between two classes, by maximizing the variance of one class while minimizing the variance of the second class (Blankertz et al., 2007b). AR models are used to describe a time-varying process with a set of coefficients that predict the next time sample. These coefficients can be used as features for EEG classification, given there are different temporal dynamics in the data classes (Pfurtscheller et al., 2002). PCA is used for unsupervised feature extraction, by mapping the data onto a new, uncorrelated space where the axes are ordered by the variance of the projected data samples along the axes, and only those axes reflecting most of the variance are maintained. Other methods search for spectral features to be used for classification (Felzer and Freisieben, 2004).

Methodologies of single-trial EEG classification algorithms have been implemented for a variety of BCI applications, using different experimental paradigms. Most commonly, single-trial EEG classification has been used for movement-based and P300 applications (Donchin et al., 2002; Wolpaw and McFarland, 2004). Movement tasks, both imaginary and real, have been studied for their potential use with disabled subjects (Müller-Gerking et al., 1999). P300 applications, based on visual or auditory oddball experiments (Donchin et al., 2002), were originally aimed at providing BCI-based communication devices for locked-in patients (Kaper et al., 2004; Sellers and Donchin, 2006; Müller et al., 2008) and can also be used for a variety of applications for healthy individuals (Sajda et al., 2010).

We aim at implementing a BCI algorithm in order to classify large image databases into one of two categories, target and non-target images. In this task, subjects are instructed to search for target images, within a rapid serial visual presentation task (RSVP; Parra et al., 2007; Bigdely-Shamlo et al., 2008; Fuhrmann Alpert et al., 2013). In this case, the goal of the classification algorithm is to automatically identify single trial spatio-temporal brain responses that are associated with the target image detection. In addition to the common challenges faced by single-trial classification algorithms for noisy EEG data, specific challenges are introduced by the RSVP task, due to the fast presentation of stimuli and the overlap between consecutive event-related responses. Some methods have thus been constructed specifically for the RSVP task.

One such method, developed by Bigdely-Shamlo et al. (2008) specifically for single-trial classification of RSVP data, used spatial Independent Component Analysis (ICA) to extract a set of spatial weights and obtain maximally independent spatio-temporal sources. A parallel ICA step was performed in the frequency domain to learn spectral weights for independent time-frequency components. PCA was used separately on the spatial and spectral sources to reduce the dimensionality of the data. Each feature set was classified separately using Fisher Linear Discriminants (FLD) and then combined using naïve Bayes fusion (i.e., multiplication of posterior probabilities).

A more general framework was proposed by Parra et al. (2007) for single trial classification, and was also implemented specifically for the RSVP task. The suggested framework uses a bi-linear spatial-temporal projection of event-related data on both temporal and spatial axes. These projections can be implemented in various ways such as linear classifiers and spatial or temporal filters (Gerson et al., 2006; Luo and Sajda, 2006; Dyrholm et al., 2007; Sajda et al., 2010). The framework has great promise in triaging image databases of natural scenes (Gerson et al., 2006), aerial images (Parra et al., 2007), and missile detection in satellite images (Sajda et al., 2010).

Our earlier work implemented a two step linear classification algorithm for RSVP tasks (Fuhrmann Alpert et al., 2013). The Spatially Weighted FLD-PCA (SWFP) algorithm first learns a spatio-temporal weights matrix that amplifies important locations for discrimination in space and time. Subsequently, it uses PCA for dimensionality reduction of the temporal domain. The final features are classified with a FLD classifier. The algorithm was tested on a RSVP task where the subject is required to detect images of a given target category out of five categories. Despite the difficult task, the algorithm achieved classification performance that is suitable for real applications.

Many of the BCI algorithms, including those described above, are focused on linear algorithms. Linear methods are simple and fast to train due to their linear constraint which makes them more robust to overfitting. This usually makes linear methods a good choice when dealing with noisy data, such as single trial EEG. On the other hand, the linearity limits the features these algorithms can learn and thus their classification performance is also limited, when the data is not linearly separable. In contrast, non-linear methods can model a wide variety of functions and thus can extract more expressive features, but require careful training to avoid overfitting.

Neural networks are a nonlinear architecture for feature extraction and classification which can learn very complex patterns. Deep, multi-layered, neural networks had achieved break-through results in various tasks such as image classification (Krizhevsky et al., 2012), speech processing (Hinton et al., 2012a), and action recognition (Ji et al., 2013). These networks have shown to be able to handle large variability in the data, which make them appealing for use with EEG. Specifically, convolutional neural networks (CNNs or ConvNets; LeCun et al., 2010 appear to be a good fit for EEG data (Cecotti and Gräser, 2011). CNNs use at least one convolutional layer, where the weights of the layer are shared across the input in order to exploit statistical correlations. The weight sharing model reduces the number of parameters in the network, which makes CNNs faster to train and less prone to overfitting. In addition, the convolution can naturally handle 2D data, as opposed to standard neural networks which operates on one dimensional vectors. In the context of EEG, CNNs can use weight sharing across the time domain to extract features that are not dependent on their temporal latency. Also, the ability to handle two dimensional data makes the extracted features easy to interpret from a physiological point of view.

**Figure 1:**
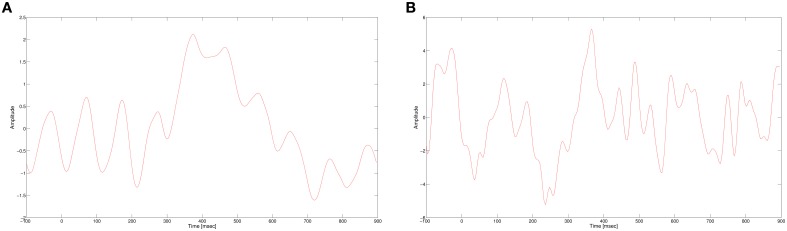
**(A)** P300 ERP at electrode Pz, computed by averaging multiple single trials. **(B)** A single trial of a target response at electrode Pz.

In this work, we use a CNN to classify single trial EEG in a RSVP task. As mentioned earlier, the P300 signal is the main discriminator between targets and non-targets for RVSP tasks. The P300 evoked response potential (ERP) is generated when a rare target stimulus is detected among non-target stimuli (Donchin et al., 2002). As its name suggests, the P300 is a positive potential, with latency around 300 ms after the stimulus onset. The ERP is usually visible when several targets responses are averaged, while in a single trial response it is masked by noise and other brain activity, as seen in Figure 1. In addition, the amplitude and latency of the P300 have a large variance between subjects and also within subjects. In spite of these problems, we expect our neural network to learn a representation of the P300 signal that would allow it to classify single trial data. The non-linearity of the neural network can potentially suggest that it would extract more expressive features than our previous algorithm, giving better classification performance.

A previous work (Cecotti and Gräser, 2011) presented a CNN for detection of P300. The suggested model has a spatial and a temporal convolutional layers, a pooling (sub-sampling) layer, and a fully connected layer. The network was tested on a P300 speller (Blankertz et al., 2004) task and shown improvement in performance.

In this paper, we build a similar neural network for a RSVP task, where the subject has to detect an image of a target category from five possible categories, while the images are presented at 10 Hz. This task has great difficulty due to the multiple categories and the fast pace of images, and our goal is to improve the classification accuracy over previous methods. We use a larger model to allow it to learn more patterns. In addition, we present a novel spatio-temporal regularization for EEG that reduces the overfitting of the model.

The architecture of the neural network is elaborated in Section 2.5 and it is evaluated on the same experiment of Fuhrmann Alpert et al. (2013). We then compare the performance of the network and investigate the learnt weights and extracted features.

## 2. Materials and methods

### 2.1. Subjects

Fifteen subjects participated in a RSVP experiment, eight females and seven males with age 26±5 years. Three subjects were excluded from the analysis due to excessive recording noise. The first excluded subject had excessive eye blink artifacts resulting in a loss of nearly 60% of the data. The second subject had technical problems with the electrodes causing repeated recording failures. The third excluded subject felt very uncomfortable, which lead to many motion artifacts, thus the subject was released half way through the experiment.

All subjects were students of the Hebrew University of Jerusalem, without any previous training in the task. All subjects had normal or corrected to normal vision, with no known neurological problems, and were free of psychoactive medications at the time of the experiment. Subjects were paid for their participation. The experiment was approved by the local ethics committee at the Hebrew university of Jerusalem, with written informed consent from all subjects. All subjects gave written informed consent in accordance with the Declaration of Helsinki.

### 2.2. Stimuli

**Figure 2:**
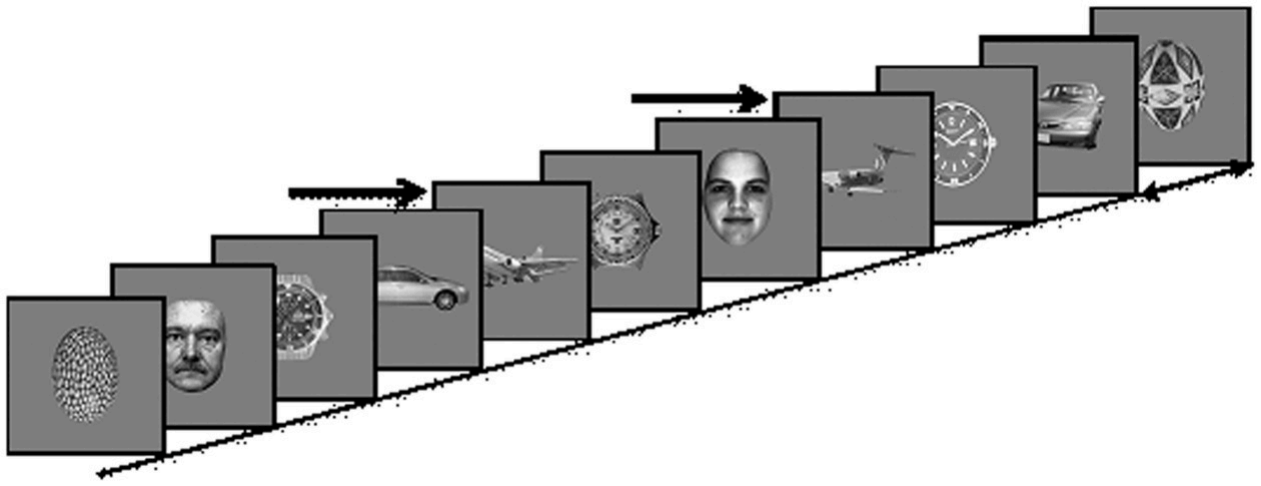
**Experimental Paradigm: Images of five different categories (Cars; Faces; Planes; Clock faces; Eggs) are presented every 90–110 ms**. In each presentation block a different object category is defined as the target category, and subjects are instructed to count the number of target occurrences, e.g., planes, marked here by arrows.

Stimuli were 360 × 360 pixels, 6.5 × 6.5 of visual angle at a distance of 100 cm, gray-scale images of five different categories including 145 exemplars each of faces, cars, painted eggs, watches, and planes, presented at the center of a CRT monitor (Viewsonic model g57f, refresh rate 100 Hz, resolution 1024 × 768) on a gray background (Figure 2). The images were preprocessed to have the same mean luminance and contrast.

### 2.3. Experimental procedure

Subjects were seated in a dimly lit, sound attenuated chamber, supported by a chin, and forehead rest. Subjects were instructed to count the occurrence of images of a predefined target category, presented within a RSVP. Each image exemplar was presented several times during the experiment. Eye position was monitored using an Eyelink 2k/1000 eye tracker (SR research, Kanata, ON, Canada) at 1000 Hz resolution. Presentation was briefly paused every 80–120 image trials and the subject was asked to report how many targets appeared in the last run, and thereafter restart the count. This was done to avoid the working memory load of accumulating large numbers.

Images were presented in four blocks, with a different target category in each block while clock faces were not used as targets. The order of blocks was counterbalanced across subjects. Each block consisted of a RSVP of 6525 images, presented without inter-stimulus intervals every 90–110 ms rates, i.e., ~10 Hz. In each block, 20% of the images were targets, randomly distributed within the block. The experimental paradigm is depicted in Figure 2.

### 2.4. EEG acquisition and preprocessing

EEG recordings were acquired by an Active 2 system (BioSemi, the Netherlands) using 64 sintered Ag/AgCl electrodes, at a sampling rate of 256 Hz with an online low-pass filter of 51 Hz to prevent aliasing of high frequencies and remove powerline noise. Seven additional electrodes were placed as follows: two on the mastoid processes, two horizontal EOG channels positioned at the outer canthi of the left and right eyes (HEOGL and HEOGR, respectively), two vertical EOG channels, one below (infraorbital, VEOGI), and one above (supraorbital, VEOGS) the right eye, and a channel on the tip of the nose. All electrodes were referenced to the average of the entire electrode set, excluding the EOG channels. Offline, a bipolar vertical EOG (VEOG) channel was calculated as the difference between VEOGS and VEOGI. Similarly, a bipolar horizontal EOG channel (HEOG) was calculated as the difference between HEOGL and HEOGR. A high-pass filter of 0.1 Hz was used offline to remove slow drifts. The data was segmented to one-second event-related segments starting 100 ms prior to and ending 900 ms after the onset of each image presentation, yielding for each subject a large spatio-temporal data matrices for the representation of single trial brain activity. Each trial data matrix was downsampled to 64 Hz to reduce computational time, as well as normalized to zero mean and variance one along each dimension. Using high sampling rates did not result any change in classification performance. Each single trial matrix consisted of 64 rows of channels and 64 columns of time samples. In each trial, the average of the time samples was removed separately for each channel (removing DC). We also re-referenced each time point to an average reference virtual electrode. Blinks were removed by rejecting trials in which the VEOG bipolar channel exceeded ±100 μV. The same criterion was also applied to all other channels to reject occasional recording artifacts and gross eye movements.

We tested the normality of each subject data matrices with Shapiro-Wilk test (Shapiro and Wilk, 1965) and Mardia's test (Mardia, 1970). Shapiro-Wilk test was performed on each dimension of the data, and rejected the null hypothesis (*p* < 0.05) of each dimension being normal. Mardia's test is a multivariate normality and thus was performed on the entire data matrix. This test also rejected the hypothesis that the data matrices are multivariate normal (*p* < 0.05). These results encourage the use of non-parametric methods, such as neural networks.

### 2.5. Neural network architecture

Our network contains three convolutional layers, two pooling layers, two fully connected layers, and an output layer.

We define a spatial convolution as a convolution along the time axis that extracts spatial filters. A temporal convolution is a convolution of a one dimensional filter along the time axis.

The input to the network is a single trial matrix of 64 channels (electrodes) by 64 time samples. The first convolutional layer performs a spatial convolution by using filters of size 64 × 1, learning features which represent a spatial distribution across the scalp. Since this layer is convolutional, the weights of the filters are shared across time and and it is insensitive to temporal latencies. The weights of this layer are regularized with a spatio-temporal penalty, described in Section 2.6.

The second layer is a max-pooling layer (LeCun et al., 2010) that reduces the dimensionality of the data. We used pooling filters in size of three samples and with stride of two samples. Therefore, we reduce the dimensionality, but we still have overlap to avoid losing too much information (Le Cun et al., 1990). The max operation provides a small invariability in the temporal domain, as long as the samples stay within the same pooling filter.

The following layers are a temporal convolutional layer, with filter size of about 100 ms, following another max-pooling layer and another temporal convolutional layer of the same size. These layers find temporal patterns in the signal that represent the change in amplitude of the spatial maps learned in the first layer. The pooling layer here mostly contributed to a faster training process. However, the convolutional layers were critical in improving the classification accuracy.

The last two layers of the network are dense, fully-connected layers, of sizes 2048 and 4096. The output layer of the network is a softmax layer of size two, which represent the probabilities of the sample to be a non-target or target sample. The input to the fully connected layers is a 1D vector constructed by concatenating the outputs of all the filters from the last convolutional layer. Therefore, these layers are sensitive to changes in time and thus it is important to use many weights here to capture the temporal variability that were not handled by the pooling layers.

**Figure 3:**
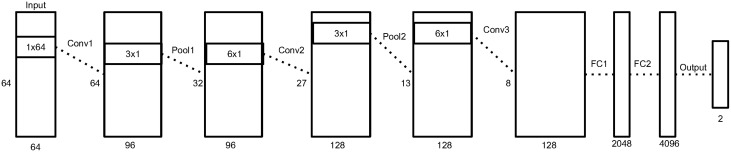
**An illustration of our neural network architecture**. Each box represent the data matrix going through the network. For the convolution and pooling layers, we note the number of time samples on the left side, and the number of output filters at the bottom. The dimensions inside the box represent the filter size of the following convolution or pooling layer. Before the first fully connected layer, the 2D data matrix is reshaped into a 1D vector. More details in Section 2.5.

Figure 3 depicts an overview of the architecture, and Table 1 shows the details of each layer in the network.

**Table 1:** **Layers sizes**.

**Layer**	**Type**	**Input size**	**Filter size**	**Number of outputs**
1	Convolution	64 time samples × 64 channels	1 × 64	96
2	Pooling	64 time samples × 96 filters	3 × 1 (stride = 2)	96
3	Convolution	32 time samples × 96 filters	6 × 1	128
4	Pooling	27 time samples × 128 filters	3 × 1 (stride = 2)	128
5	Convolution	13 time samples × 128 filters	6 × 1	128
6	Fully connected	8 time samples × 128 filters	N/A	2048
7	Fully connected	2048	N/A	4096
8	Output	4096	N/A	2

The ReLU non-linearity (Nair and Hinton, 2010), *f*(*x*) = *max*(0, *x*), is used after each convolutional and fully-connected layer, and Dropout (Hinton et al., 2012b) is used on each fully-connected layer to decrease overfitting.

The network was not constrained to specific temporal or spatial regions in the data. Although we know that our main target signal is the P300, the network might learn other good features that can assist classification, as occurred in Fuhrmann Alpert et al. (2013).

The implementation of this architecture was based on Caffe Jia et al. (2014) and was trained on a NVIDIA GTX 650 GPU. The parameters of the training process are described in Section 2.7.

### 2.6. Spatio-temporal regularization

Single trial EEG is highly variable, even within the same subject, as the EEG contains interferences from ongoing brain activity and measurement noise. These types of noise suggest the use of regularization in order the keep the weights of the network small to reduce overfitting. The common regularization methods for neural networks are L2 and L1, which add a penalty to the weights according to their magnitude and sign. Here we suggest a new regularization, which takes into consideration the structure of EEG, and enforces a stronger regularization in time points that change sharply.

We introduce a spatio-temporal penalty specifically for EEG. We make the assumption that our target signal is changing slowly compared to the noise in the data. The P300 ERP evolves in time over a period of 200–300 ms and occupies frequency bands up to 10 Hz (Kolev et al., 1997), similarly to other ERPs. On the other hand, noise is often rapidly changing with high frequency components. Therefore, we encourage our first convolutional layer to learn spatial filters that change slowly in time by adding a penalty to the cost function. The penalty term regularizes the spatial filters such that the filters activations will be smooth, encouraging smaller differences between consecutive temporal values. This regularization affects both spatial weights learned by the network, and the temporal activations given by first convolutional layer, and thus we call it a spatio-temporal regularization. The penalty term is

(1) $$\begin{array}{l} L_{p}=\lambda_{p}\overset{N-1}{\underset{t=1}{\sum }}{\left(a_{t+1}^{l}-a_{t}^{l}\right)}^{2} \end{array}$$

where $a_{t}^{l}$ is the convolution output at time *t* in layer *l*, and λ_*p*_ is the regularization coefficient. We derive the penalty term with respect to the convolution weights *W*,

(2) $$\begin{array}{l} \frac{\partial L_{p}}{\partial W}=\frac{\partial L_{p}}{\partial a_{t}^{l}}\frac{\partial a_{t}^{l}}{\partial W} \end{array}$$

The first gradient is computed by

(3) $$\begin{array}{l} \frac{\partial L_{p}}{\partial a_{t}^{l}}=\left\{ \begin{array}{cc} -2\left(a_{2}^{l}-a_{1}^{l}\right)\; & t=1\\ 2\left(a_{n}^{l}-a_{n-1}^{l}\right)\; & t=n\\ 4a_{t}^{l}-2a_{t-1}^{l}-2a_{t+1}^{l}\; & t\neq 1,n \end{array}\right. \end{array}$$

For the second gradient, we note that $a_{t}^{l}$ is computed by a convolution of the weights with the output of the previous layer,

(4) $$\begin{array}{l} a_{t}^{l}=W*a_{t}^{l-1} \end{array}$$

if we derive $a_{t}^{l}$ with respect to weight *W*_*i, j*_, we simply have

(5) $$\begin{array}{l} \frac{\partial a_{t}^{l}}{\partial W_{i,j}}=E_{i,j}*a_{t}^{l-1} \end{array}$$

where *E*_*i, j*_ is a matrix of zeros with a single value of one in position (*i, j*).

**Figure 4:**
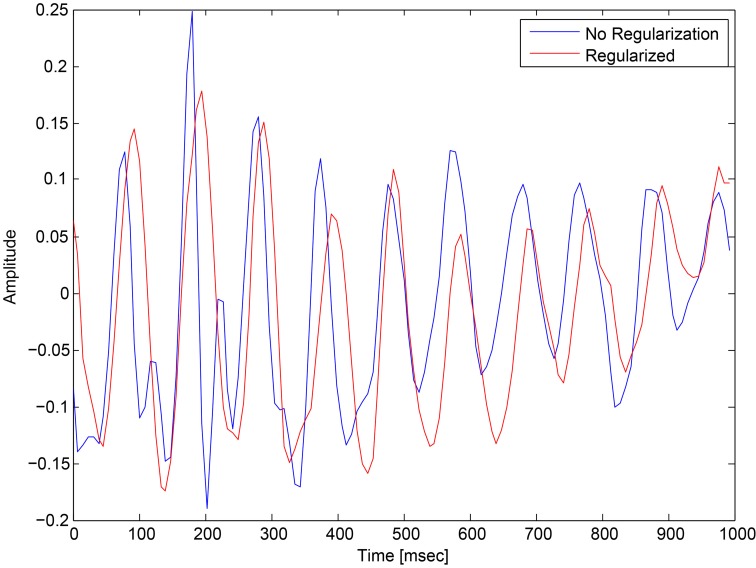
**Comparison of the activations of a convolutional layer with the spatio-temporal regularization (red) and without (blue)**.

**Figure 5:**
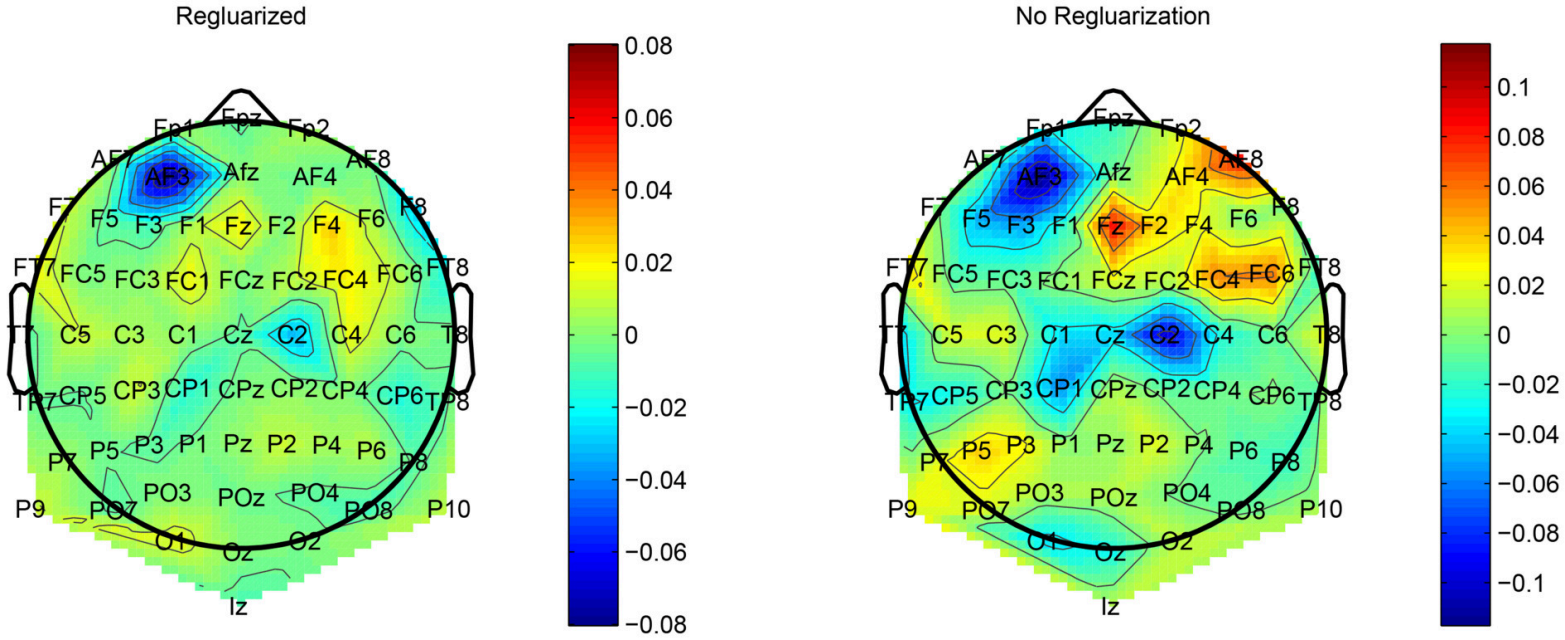
**Comparison of the weights of a convolutional layer with the spatio-temporal regularization (left) and without (right)**.

This regularization reduced the overfitting in our network and contributed to an improved classification performance. In addition, it had an obvious impact on the convolution filters by making them more smooth and more stable in time. To demonstrate the effect of this regularization, we ran a simple network with a small number of spatial convolution filter and high coefficient for the spatio-temporal penalty term. Figure 4 shows a comparison of the activations of the regluarized convolutional layer with and without the regularization. We can see that the regularized activations are smoother with slower changes in the amplitude. Figure 5 shows the effect of the regularizer on the trained filters of the convolutional layer. Since the weights of this layer represent the EEG channels, we treat it as a spatial distribution across the scalp and plot on a head-plot using EEGLAB (Delorme and Makeig, 2004). We can see that the general shape of the distribution is similar but the regularized plot has softer peaks.

### 2.7. Learning parameters

The network is trained by minimizing the multinomial logistic regression loss function:

(6) $$\begin{array}{l} L=-\overset{N_{samples}}{\underset{i}{\sum }}\log\left[\left(1-y^{\left(i\right)}\right)h_{0}\left(x^{\left(i\right)}\right)+y^{\left(i\right)}h_{1}\left(x^{\left(i\right)}\right)\right] \end{array}$$

where *N*_*samples*_ is the number of training samples, *x*^(*i*)^ is the *i* training sample, *y*^(*i*)^ is the true label of sample *i* and *h*_*k*_ is the neural network output unit *k*. The loss function is minimized with SGD, with learning rate of 0.001 and 0.9 momentum (Polyak, 1964). The momentum update *V* is computed with

(7) $$\begin{array}{l} V=\gamma V+\eta Q \end{array}$$

where η is the learning rate, γ is the momentum coefficient and *Q* is the gradient of the loss function with respect to the network parameters. The parameters *W* are then updated with

(8) $$\begin{array}{l} W=W-V \end{array}$$

The gradient is computed with the classical back-propagation algorithm.

It should be noted that computing the gradient using a single sample, instead of using a mini-batch, accelerated the convergence time of the network. The parameters of the network were chosen empirically by cross-validation.

### 2.8. Class imbalance

The nature of our experiment causes the data classes to be imbalanced, as only 10% of the single trials are targets. Gradient descent methods do not perform well on unbalanced datasets because the gradient follows the majority class. To overcome this bias, we bootstrapped the targets class to match the size of the non-targets, only in the training set. Although this caused some overfitting on the target class, it provided a more balanced classification performance in our experiments.

## 3. Results

### 3.1. Classification performance

We tested our network using a random cross-validation procedure. The dataset was randomly split into a 80% training set and a 20% test set repeatedly. The presented results are an average of the test performance of ten runs on random train/test splits.

The network was tested on each subject separately, using the concatenation of all four blocks per subject. Therefore, the network must learn a general target response and not a specific response to one of the categories, e.g., N170 for faces (Bentin et al., 1996).

Classification results are summarized in Table 2. The performance is described in terms of correctly classified trials, hit rate (true positive rate) and false alarm rate (false positive rate), where positive is the target class. The correct classification metric is defined as the sum of correct positives and negatives among all samples, and thus can show a distorted view of performance due to the imbalanced classes in our data set; if we classify everything as non-target, we get 90% correct classification. Therefore, we also compute the Area Under the Curve (AUC) and balanced accuracy metrics. The balanced accuracy is defined as (true positive rate+true negative rate)∕2. The standard deviations in the table and figures are across the repeated cross validation test sets, while the deviations at the mean row is across subjects.

**Table 2:** **Classification performance**.

**Subject**	**Correct**	**Hits**	**False alarms**	**AUC**	**Balanced accuracy**
501	77.6±1.9	68.7±2.1	21±2	0.81±0.008	73.8±0.8
502	75.4±1.3	64.5±1.6	23.2±1.4	0.77±0.005	70.6±0.8
503	73.8±1.2	62.3±1.3	24.6±1.5	0.75±0.01	68.8±0.9
504	83.1±1.1	73±1.7	15.5±1.4	0.86±0.007	78.7±0.6
506	70±1.3	59.1±1.6	28.8±1.4	0.70±0.009	65.1±1
507	76.8±1.2	64.8±1.6	21.8±1.6	0.78±0.009	71.4±0.8
508	70.8±1	60.2±1	27.8±1.2	0.72±0.008	66.2±0.4
510	71.2±1.3	60.4±2.4	27.3±1.6	0.72±0.01	66.5±1
511	69.8±1.1	56.5±2	28.4±1.5	0.69±0.009	64±0.7
512	73.7±1.3	65.2±1.7	25.1±1.5	0.76±0.008	70±0.6
513	82.3±1.1	74.6±1.9	16.6±1.2	0.86±0.005	79±0.7
515	74.6±1.7	63.7±1.1	23.9±2.1	0.76±0.008	70±0.5
Mean	75±4.4	64.4±5.47	23.7±4.36	0.77±0.05	70±4.86

**Figure 6:**
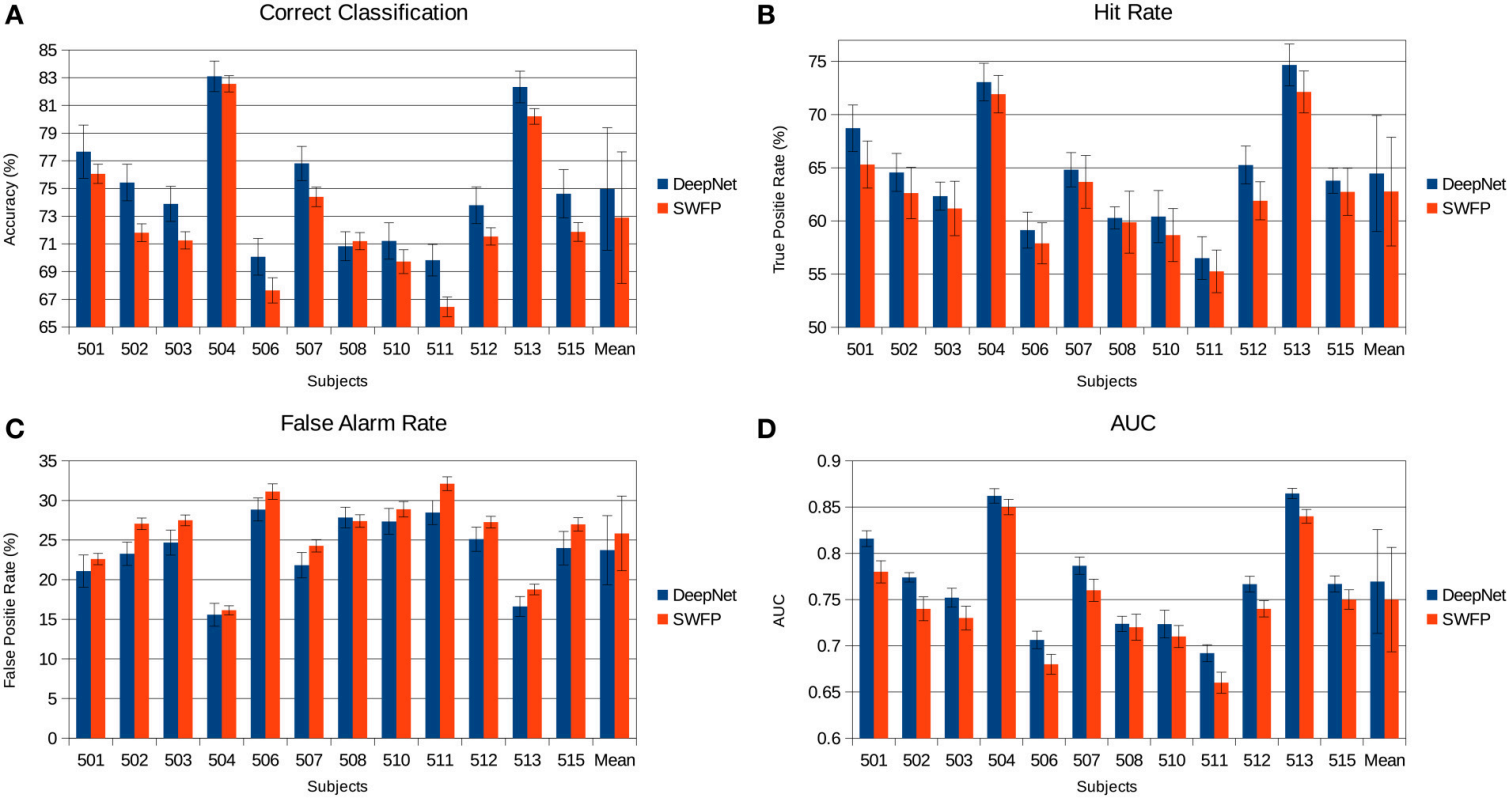
**Comparison of the neural network performance and SWFP (Fuhrmann Alpert et al., 2013) performance on the same task and dataset**. **(A)** Correct classification across subject. **(B)** Hit rate (true positive rate). **(C)** False alarm rate (false positive rate). **(D)** Area under the curve (AUC) of the ROC graph.

The various metrics in Table 2 show the classification performance is well above chance, although it does vary across subjects. Correct classification ranges from 70.8 to 83.1% while balanced accuracy ranges from 66.2 to 79%. Figure 6 compares the performance of the neural network to the SWFP algorithm (Fuhrmann Alpert et al., 2013). The comparison was performed on the same random cross-validation splits for both algorithms. We can see consistent improvement of performance across all subjects, except for 508 where the two algorithms perform about the same. On average, the balanced accuracy has improved by 2% when using the neural network. In addition, the non-parametric Wilcoxon signed rank tests (*p* < 0.05) show that the neural network does significantly better than the SWFP on all presented metrics.

### 3.2. Features analysis

**Figure 7:**
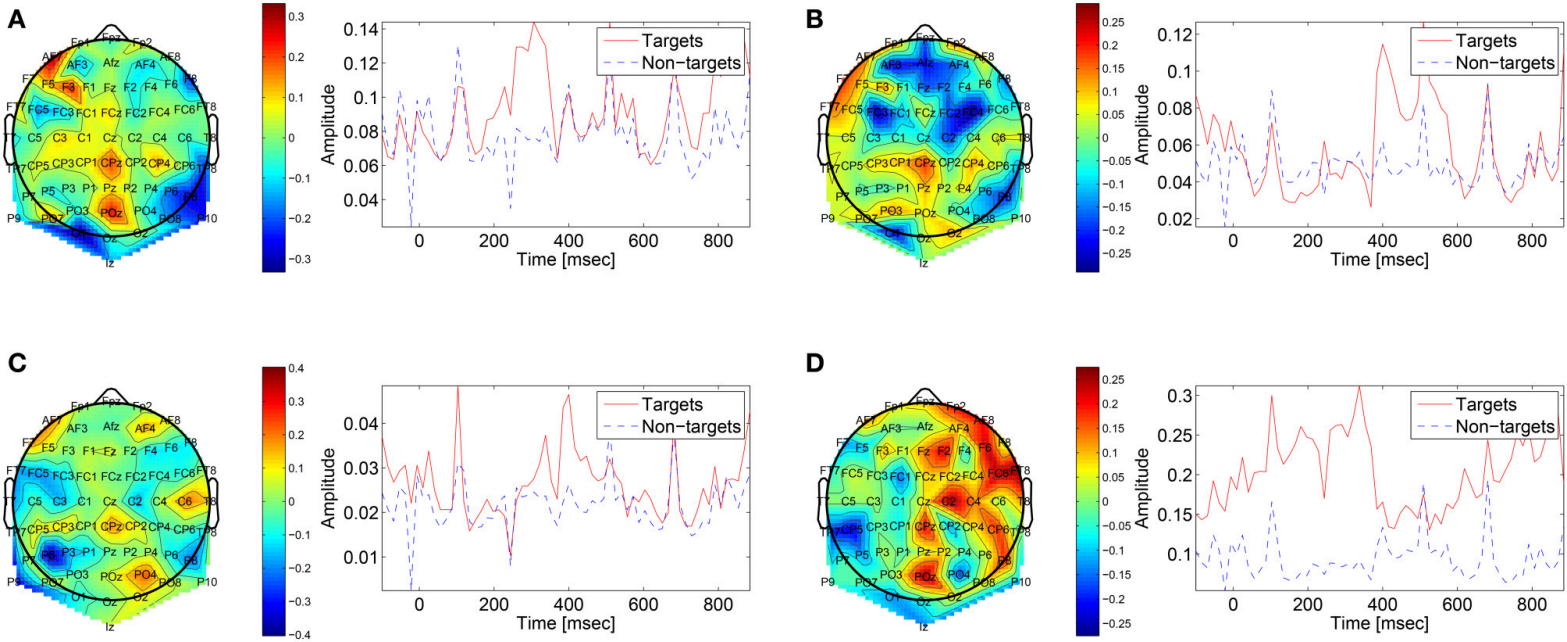
**Representative spatial filters and their temporal features from a sample subject (504)**. The left side shows the spatial filters distributed on the scalp. The right side shows the mean temporal activations of the corresponding spatial filter where red is for targets samples and blue is for non-targets trials. We can see that the spatial maps are similar to the spatial distribution of the P300 with a high amplitude at central-parietal electrodes.

**Figure 8:**
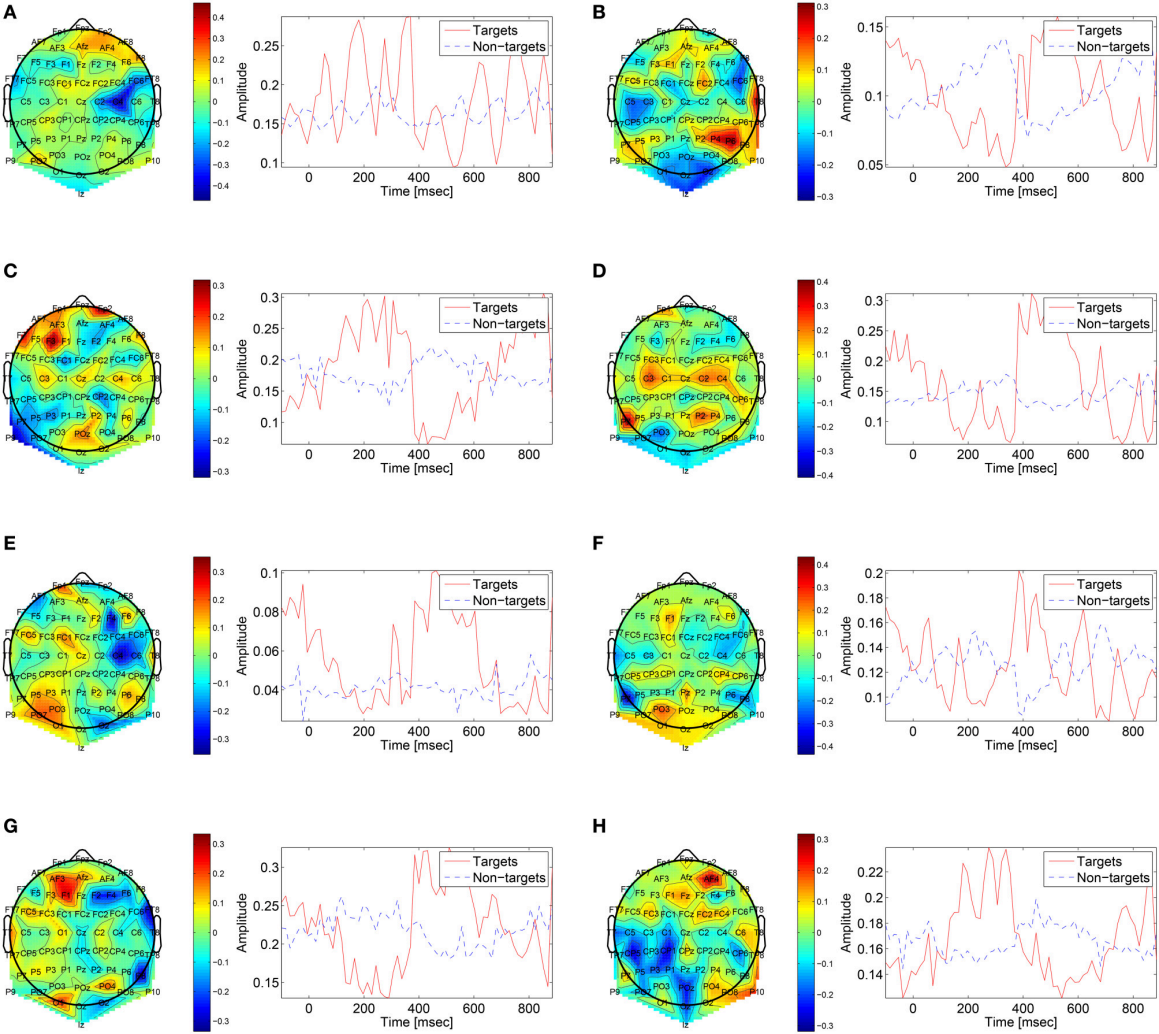
**Sample of spatial filters and temporal activations for subjects: (A) 501, (B) 502, (C) 503, (D) 507, (E) 508, (F) 511, (G) 512, (H) 513**.

The first convolutional layer has filters in size 64 × 1 so it extracts features only from the spatial domain. The filters are weights for the EEG electrodes and this can be thought of as spatial filters that represent the amplitude distribution of the signal across the head. Figure 7 shows a sample of these filters along with their output features, aggregated over the target and non-target trials of a sample subject (504). The spatial maps show relatively high amplitude at central-parietal and frontal electrodes. This is a typical distribution for the P300 (Polich, 2007). In map (Figure 7A) we also see a high occipital weight magnitude, which is known to be related to the visual system. The features of the spatial maps can be thought of as the temporal amplitude of the spatial map in time. We can see that there are peaks of activity in 300–400 ms, which is similar to the P300 activation patterns. However, some maps show high amplitude as early as 50 ms and as late as 850 ms. These might be explained by the overlapping nature of our experiment. Recall that the images are presented at 10 Hz and each single trial is recorded for one second, which means that one single trial contains responses to multiple images and thus the latencies are aligned to different images than the one displayed at time zero. Figure 8 shows a sample of filters and activations from other subjects as well.

### 3.3. Benchmark results

We validate our neural network on a public data set from the third BCI competition (Blankertz et al., 2004). The data contains the results of a P300 speller experiment from two subjects. In this experiment, the subject focused on one of 36 characters on a screen. The characters are arranged in a 6 × 6 matrix where each row and column is separately intensified in a random order. When the row or column of the desired character is highlighted, a P300 ERP should be generated. Thus, we can detect which character the user selected by detecting the P300 signal for each row and column and selecting the appropriate cell in the matrix. Please see data set IIb in Blankertz et al. (2004) for more details.

In the described experiment, each row and column was highlighted several times to allow the algorithm to use the repeated trials to increase the SNR and the character recognition rate. Here, however, we are interested plainly in detecting the P300 signal and therefore we treat each response as a single trial and we do not use the repeated responses, similarly to our RSVP experiment.

We used the same neural network described above for this data set. The network was trained and tested on the data from Blankertz et al. (2004). The data was preprocessed to have zero mean and variance one. For subject A, the network achieved 71.9% correct classification, 68.13% hit rate, and 27.36% false alarm rate. For subject B, 79.1% correct, 70% hit rate, and 19.11% false alarm rate. This performance is slightly better than presented in Cecotti and Gräser (2011) for the P300 detection task, which achieved 70.5% correct, 67% hit rate, and 29.5% false alarm rate for subject A, and 78.19% correct, 69% hit rate, and 20% false alarm rate for subject B^1^. Although we did not tune our network for this data set, it still outputs valid results and shows that this is a robust model.

### 3.4. Cross-session variability

EEG is known to be non-stationary, especially between sessions of recordings (Blankertz et al., 2007a). Therefore, it is important to test any EEG classification algorithm on data where the training and validation sets were recorded on different sessions. We test our neural network on a similar experiment to the one described above, only this time the subject was asked to identify the same target (cars) throughout the experiment. The subject performed two sessions of the experiment, on different days. We use the first day as a training set and the second day as a validation set, and compare performance with using only on the data from the second day both for training and validation. The results are presented in Table 3 when using the neural network and SWFP. We see that the performance of the neural network surpasses SWFP. In addition, the difference between the balanced accuracy of same session vs. cross-session is small in the neural network compared to SWFP. With the neural network, the differences are 4.62, 1.97, and 3.93 for subjects 701, 702, and 703, respectively. With SWFP, the differences are 6, 2.12, and 6.11.

**Table 3:** **Cross-session performance**.

**Method**	**Subject**	**Training day**	**Testing day**	**Correct**	**Hits**	**False alarms**	**Balanced accuracy**
Neural network	701	Day 1	Day 2	91.15	81.6	7.7	86.95
Neural network	701	Day 2	Day 2	93.84	88.66	5.52	91.57
Neural network	702	Day 1	Day 2	95.31	86.64	3.62	91.51
Neural network	702	Day 2	Day 2	95.59	90.77	3.81	93.48
Neural network	703	Day 1	Day 2	90.91	83.68	8.19	87.74
Neural network	703	Day 2	Day 2	92.14	91.07	7.72	91.67
SWFP	701	Day 1	Day 2	91.51	76.27	6.61	84.83
SWFP	701	Day 2	Day 2	93.26	87.72	6.05	90.83
SWFP	702	Day 1	Day 2	93.15	87.3	6.12	90.59
SWFP	702	Day 2	Day 2	95.17	89.55	4.12	92.71
SWFP	703	Day 1	Day 2	89.83	78.67	8.77	84.95
SWFP	703	Day 2	Day 2	94.55	86.59	4.45	91.06

## 4. Discussion

BCI applications are required to solve a hard problem - decoding the brain electrical activity into a meaningful signal, dealing with high variability and non-stationary noise. These difficulties lead to the use of ML algorithms to solve BCI applications. In this case, the learning algorithm has to be trained in order to learn the properties of the P300 ERP for a specific subject in order to later detect it in an unknown trial. Several algorithms have been previously introduced for this task (Gerson et al., 2006; Parra et al., 2007; Bigdely-Shamlo et al., 2008). In general, most of these algorithms produce a linear mapping of the single trial EEG into a feature space, which are then classified. The mapping of the data to features is usually constructed manually, while the parameters of the mapping are learned. For example, with SWFP (Fuhrmann Alpert et al., 2013) we decided upon learning a spatio-temporal weights matrix as a first step, and a second step where temporal features are extracted per channel and then classified. All of these choices make sense in the context of our application, but were strictly chosen in a manual, handcrafted, process.

Recent works on neural networks suggest a different approach - using the same basic module for feature extraction and classification. The neural network is a generic architecture with several building blocks that can be built for a wide variety of applications such as object recognition in images and speech recognition (Krizhevsky et al., 2012; Hinton et al., 2012a). We take advantage of this architecture by constructing a neural network for EEG, which is not entirely different from a neural network for images. This a significant advantage as we can spend less effort on hand-tuning the feature extraction process. Neural networks do require tuning of hyper-parameters, such as the learning rate, number of layers, number of neurons and even allow greater customization like the spatio-temporal penalty introduced above. Alternatively, building a new method to extract features contain endless options as many algorithms can be combined in an infinite number of ways. Although this route can sometime lead to interesting features and algorithms, it is not clear that it is the most efficient option when we want to maximize the classification performance.

Here, we present a CNN for feature extraction and classification of a complicated RSVP task where a subject has to detect a target image within five possible categories. Similar works (Gerson et al., 2006; Parra et al., 2007; Bigdely-Shamlo et al., 2008) experimented with RSVP tasks with two categories. Even with the increased difficulty of five categories, the neural network succeeds in detecting the P300 and surpass the classification accuracy of our previous algorithm which was manually designed for this application. In addition, we test our network on cross-session data, where the training and validation sets were recorded on different days. Also in this scenario, the network surpasses the performance of SWFP, suggesting that it learns more robust features that are stable even across sessions.

Within our neural network, the convolutional layer makes a good fit for EEG as it efficiently learns features across the time domain. This is important when dealing with EEG since it is hard to predetermine the latency of the target signal which we want to learn. In practice, one can say that the convolutional layers in the network learn spatial and temporal features without considering their temporal position. On the other hand, the fully connected layers do enforce a temporal structure by learning a specific weight for each time point.

Although the network has shown improved performance compared to Fuhrmann Alpert et al. (2013), there are several drawbacks of using a neural network. Mainly, the training time of a network: on this dataset, it took up to thirty minutes to train a neural network, while training SWFP takes no more than two minutes. On the other hand, developing a new algorithm can take a considerable amount of time and effort. Using a neural network has less design choices and existing models can be borrowed from other domains, e.g., vision and speech.

## 5. Conclusion

We showed that deep neural network models are an effective tool for single trial P300 classification. Even for a difficult RSVP task with five categories, we achieve impressive classification performance that surpasses our earlier work. The neural network model is regularized with a novel spatio-temporal regularizer, which encourages the network to learn smooth features, as shown in Figures 4, 5 and thus reduces overfitting to noisy samples. These results should encourage us to keep pursuing the use of neural networks in other EEG tasks.

Although the network was not constrained to specific regions in time and space, we showed that it extracts meaningful features from the EEG. The learnt features and their activations show correlation with the known P300 ERP both in time latency and electrode location.

The main advantage of a neural network is that it doesn't require any prior assumptions on the data, making it suitable for many classification problems, without manual engineering of complicated feature extraction algorithms. This is a huge advantage that can save significant research and development time while obtaining state of the art results in classification tasks. In addition, it has been shown in other domains that deep networks yield improved performance as the training data grow. Therefore, we should look at building large data sets for EEG which will allow to explore larger and deeper models.

### Conflict of interest statement

The authors declare that the research was conducted in the absence of any commercial or financial relationships that could be construed as a potential conflict of interest.

## Footnotes

^1^These results were the best out of Tables 2, 3 in Cecotti and Gräser (2011)
